# Efficacy and Safety of Omega-3 Fatty Acids in Ameliorating Pruritus in Adult Patients With Chronic Kidney Disease: A Meta-Analysis of Randomized Controlled Trials

**DOI:** 10.7759/cureus.66452

**Published:** 2024-08-08

**Authors:** Saad Alqahtani, Basel H Bakhamees, Fahad M Almalki, Aseel B Alshaer, Abdullah F Altaymani, Maha M Alazmi, Khadijah A Almutlaq, Ahmed M Albalawi, Alanoud A Alfaqih, Raghad Abdullah, Abeer H Alnashri, Amina M Ebrahim, Juri Alghofaili

**Affiliations:** 1 Family Medicine, King Salman Armed Forces Hospital, Tabuk, SAU; 2 Faculty of Medicine, King Abdulaziz University, Jeddah, SAU; 3 Medical School, King Abdulaziz University, Jeddah, SAU; 4 Internal Medicine, King Khalid University Hospital/King Saud University Hospital, Riyadh, SAU; 5 Emergency Medicine, Al Jouf University, Sakaka, SAU; 6 Medical School, Al Jouf University, Sakaka, SAU; 7 Medical School, King Faisal University, Al Hasa, SAU; 8 Medical School, Tabuk University, Tabuk, SAU; 9 Medical School, Imam Mohammad Ibn Saud Islamic University, Riyadh, SAU; 10 Department of Clinical Nutrition, Faculty of Applied Medical Sciences, Umm Al-Qura University, Makkah, SAU; 11 General Practice, Al-Qunfudah General Hospital, Makkah, SAU; 12 General Practice, Ain Shams University, Cairo, EGY; 13 Medical School, Qassim University, Buraydah, SAU

**Keywords:** chronic kidney disease, uremic pruritus, uremia, omega-3 fatty acids, meta-analysis

## Abstract

Chronic kidney disease-associated pruritus (CKD-aP) represents a common distressing problem in patients with end-stage renal disease. This study aimed to assess the efficacy and safety of omega-3 supplementation in the treatment of CKD-aP. MEDLINE/PubMed, Cochrane Central Register of Controlled Trials, Web of Science, ProQuest, and Scopus databases were searched systematically for articles published from inception until May 21, 2024. Outcomes were pruritus severity at the end of the study or its change from baseline (primary) and intervention-related adverse effects (secondary). Results were pooled as standardized mean difference (SMD) and risk ratio (RR) for numeric and dichotomous outcomes, respectively, along with their 95% confidence intervals (CIs). Eight studies were included. Treatment with omega-3 fatty acids showed a significantly lower severity of CKD-aP at the end of treatment (pooled SMD (95% CI) = -1.03 (-1.85, -0.22), p = 0.024) and changed from baseline (pooled SMD (95% CI) = -0.93 (-1.57, -0.28), p = 0.014). Omega-3 supplementation reduced the risk of CKD-aP (pooled RR (95% CI) = 0.68 (0.12, 3.81), p = 0.661). Omega-3 fatty acid supplementation appears to be a promising effective and safe treatment for CKD-aP. However, the included studies had several limitations that warrant further high-quality studies to elucidate its effect and investigate the causes of non-response in patients who did not improve.

## Introduction and background

A considerable proportion of patients with end-stage renal disease (ESRD) suffer from itching which may become exacerbated at night. The condition is known as chronic kidney disease-associated pruritus (CKD-aP) or uremic pruritus [1]. CKD-aP negatively impacts the quality of life in patients with ESRD. Patients with CKD-aP suffer from considerable discomfort, which leads to experiencing anxiety and depression as well as disturbing sleep and causing chronic fatigue [2,3]. All of these effects can also lead to a negative impact on the mental and physical health of the patients [4]. Moreover, patients with severe CKD-aP are more likely to abandon or miss dialysis sessions [5]. The presence of CKD-aP increases the risk of using more medications (e.g., intravenous antibiotics, erythropoiesis-stimulating drugs), infections, hospitalization, and mortality [5,6].

The prevalence of CKD-aP is estimated to be 15%-55% in pre-dialysis patients but rises to 50%-90% in patients on hemodialysis or peritoneal dialysis [2,7].

The exact etiology of CKD-aP is still not well defined, despite the assessment of several potential mechanisms. The pathogenesis seems to involve the interaction of several uremic and non-uremic factors. Moreover, CKD induces a systemic pro-inflammatory state that may cause inflammation of nerves and skin [8]. The risk of developing CKD-aP increases in patients with increased serum calcium, phosphorus, aluminum, magnesium, urea, ferritin, and β2-microglobulin; skin xerosis; anemia and erythropoietin insufficiency; hypervitaminosis A; low levels of albumin and transferrin; secondary hyperparathyroidism; systemic inflammation; and uremic neuropathy [4,9,10].

Unfortunately, CKD-aP may not respond to conventional antipruritic treatments [11]. Several lines of treatments have been assessed, both non-pharmacologic and pharmacologic modalities. Non-pharmacologic modalities of treatment included regular efficient dialysis, the use of non-complement-activating dialysis membranes, dietary restrictions, acupuncture, and ultraviolet B therapy. Pharmacological modalities included emollients, topical corticosteroids, endocannabinoids, and tacrolimus, as well as the administration of antihistamines, gabapentin, naltrexone, pentoxiphylline, cholestyramine, epoetin, or ketotifen [11].

Previous studies found that the serum levels of several essential fatty acids (e.g., omega-3) are significantly lower in ESRD patients on hemodialysis compared to healthy controls [12-14]. The reduction in essential fatty acids was hypothesized to cause or aggravate the pro-inflammatory state and CKD-aP in ESRD patients [15]. There are three main omega-3 fatty acids, namely, α‑linolenic acid (ALA), eicosapentaenoic acid (EPA), and docosahexaenoic acid (DHA), which are long-chain, polyunsaturated, essential fatty acids. As their production inside the human body is limited, dietary intake or supplementation is the principal route for obtaining those essential fatty acids in humans [16]. The EPA and DHA are obtained mainly from marine sources, while ALA is obtained mainly from some seeds, nuts, and vegetable oils [17].

Omega-3 fatty acids, and the derived eicosanoids, showed several benefits in patients with cardiovascular disease, diabetes [18], cancer [19], and nephropathies [20]. However, omega-3 supplementation is not currently routinely prescribed in CKD patients, despite their potential positive effects in such patients. Previous studies reported that the administration of oral supplementations can significantly increase serum levels of omega-3 fatty acids [21] and thereby may decrease the severity of CKD-aP [12,13,22]. However, evidence is still lacking regarding the efficacy of omega-3 supplementations in alleviating the severity of CKD-aP. Therefore, this systematic review and meta-analysis aimed to assess the efficacy and safety profile of omega-3 supplementation in the treatment of CKD-aP.

## Review

Methodology

This systematic review and meta-analysis followed the principles of the Cochrane Handbook for Systematic Reviews of Interventions, version 6, and the Preferred Reporting Items for Systematic Reviews and Meta-Analyses (PRISMA) guidelines [23].

Eligibility Criteria for the Included Studies

Patients diagnosed with CKD and suffering from CKD-aP.

Intervention

Oral administration of omega-3 fatty acid supplementation.

Comparator

Placebo or conventional treatment.

Outcomes

The studies needed to assess and report the severity of CKD-aP as measured by the visual analog scale (VAS) or any other validated scale.

Types of Studies

Randomized controlled clinical trials (RCTs) published in English from inception until May 21, 2024, were eligible for inclusion.

Exclusion Criteria

We excluded conference abstracts, duplicate records, case reports, observational studies, non-randomized studies, review articles, commentaries, editorials, clinical guidelines, and studies without a control group.

Search Strategy

We conducted an online search on Medline/PubMed, Cochrane Central Register of Controlled Trials (CENTRAL), Web of Science, ProQuest dissertation and theses, and Scopus. No search filters were used. The search terms for Medline/PubMed included ((“fish oil”[Text Word] OR “omega 3”[Text Word] OR “fatty acids”[Text Word] OR “fish oils”[MeSH Major Topic] OR “fatty acids, omega 3”[MeSH Major Topic]) AND (“chronic renal failure”[Text Word] OR “chronic kidney disease”[Text Word] OR “end stage renal disease”[Text Word] OR “dialysis”[Text Word] OR “uremia”[Text Word] OR “uremic”[Text Word] OR “renal insufficiency, chronic”[MeSH Major Topic]) AND (“pruritus”[Text Word] OR “itch*”[Text Word] OR “pruritus”[MeSH Major Topic])). The Polyglot Search Translator [24] from Systematic Review Accelerator (SRA), Bond University, was used for the formulation of the search terms for the four databases.

Selection of Studies

Two independent reviewers conducted the online search, screening the titles and abstracts, and assessing the full text of relevant records. Any disagreements between the two reviewers were settled by consulting a third reviewer.

Data Extraction

Extracted data included (a) the study design, eligibility criteria of individual studies, and sample size; (b) the regimen of omega-3 fatty acid supplementation and the used control; (c) patients’ age, sex, and baseline severity of CKD-aP; and (d) the outcomes, i.e., severity of pruritus post-intervention and/or the change from baseline as well as any observed intervention-related adverse effects.

Measured Outcomes

Primary outcome: Efficacy of the intervention in improving CKD-aP measured by comparing post-intervention and/or change from baseline in any score measuring pruritus severity.

Secondary outcome: The safety of omega-3 fatty acid supplementation was assessed by comparing the observed intervention-related adverse events.

Assessment of the Risk of Bias in Included Studies

The risk of bias (ROB) in the included trials was assessed using the ROB2 tool for randomized clinical trials [25]. The tool consists of five domains which assess the risk of introducing bias in the processes of randomization, adherence to the assigned treatment, missed data, measurement of the outcome, and reporting of the outcomes. The overall ROB was identified by selecting the highest level of ROB in the five domains.

Data Synthesis

The extracted data from the included studies were summarized as tables in the systematic review. Pooling of the results was performed with the R Statistical language (version 4.4.0) [26] using the packages meta (version 7.0.0) [27] and dmetar (version 0.1.0) [28]. As the severity scores for assessing the severity of CKD-aP varied across the studies, we used the standardized mean difference (SMD) to present and pool the results. The risk ratio (RR) was calculated for the risk of CKD-aP post-intervention which was a categorical outcome. Significant heterogeneity was detected if the p-value from the Cochran chi-square test was <0.1 and/or the I2 index was ≥50%; in this case, a random-effects model was used for pooling the studies’ results. Otherwise, a fixed-effect model was used [29]. A p-value below 0.05 was selected to indicate statistical significance in the tests comparing the intervention and control groups. No tests were performed to assess publication bias as the number of included studies was less than 10.

Results

Results of the Literature Search and Study Selection

**Figure 1:**
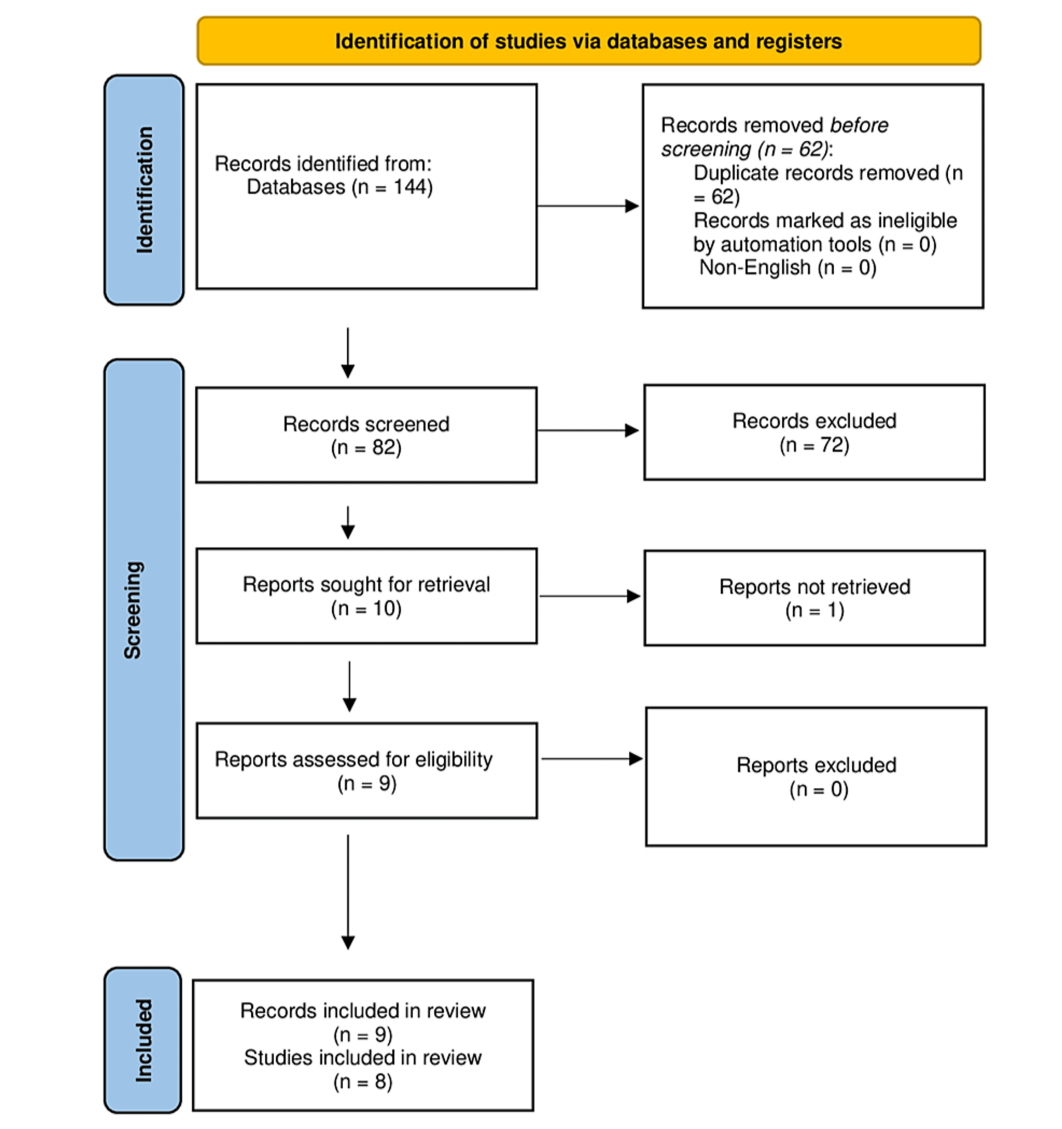
Preferred Reporting Items for Systematic Reviews and Meta-Analyses flowchart showing the results of the literature search and study selection.

The search strategy yielded 144 records, of which 62 were duplicates and were removed. The remaining 82 records underwent screening of their titles and abstracts, and 72 records were excluded. The full texts of the remaining 10 records were sought, but the full text of one record was not retrieved [30]. The full texts of the other nine records were obtained and were eligible for inclusion in this systematic review and meta-analysis [12,13,31-37]. Two records were from the same study, published as a thesis [12], and then as a journal article [13]. Ultimately, nine records from eight studies were included in this systematic review and meta-analysis (Figure 1).

Basic Characteristics of the Included Studies

All included studies were RCTs. Two studies were conducted in the United States [12,13,31], while the other six studies were conducted in Iran [32-37]. Patients in all studies were adults with CKD. The eligibility criteria for the individual studies showed a few variations, as some studies included only those on hemodialysis and excluded peritoneal dialysis. Other studies stated some eligibility criteria based on laboratory cutoff limits for some investigations such as hemoglobin and phosphorus levels (Table 1).

**Table 1:** Characteristics of the included studies (n = 8). CRF: chronic renal failure; DM: diabetes mellitus; ESRD: end-stage renal disease; GIT: gastrointestinal tract; HD: hemodialysis; INR: international normalized ratio; KT/V: K is the urea clearance (mL per minute), T is the treatment duration (minutes), and V is the volume of urea distribution (mL); NSAIDS: non-steroidal anti-inflammatory drugs; PD: peritoneal dialysis; PTH: parathyroid hormone

Study	Location	Inclusion criteria	Exclusion criteria
Peck et al. [12,13]	United States	ESRD patients	DM; age <18 or >78 years; beta-adrenergic blocking drugs or L-carnitine; malabsorption or other conditions affecting fatty acid metabolism; concurrently involved in other research studies
Begum et al. [31]	United States	Patients on maintenance HD of both sexes; age ≥20 years, with symptoms of dry and/or itchy skin	Patients with DM, malabsorption problems, or other conditions that may affect fatty acid metabolism
Ghanei et al. [32]	Iran	Patients with ESRD who were under intermittent HD and had pruritus for >3 months with no response to antipruritic drugs	Pruritus because of other skin diseases; malignancies; hepatic cholestasis; hepatitis B and C; steroid treatment; hemoglobin <10g/dL and KT/V index <1.2; warfarin treatment; allergy to fish oil
Lahiji et al. [33]	Iran	Age >18 years undergoing CAPD for ≥1 month complaining of pruritus for >8 weeks	Poor compliance; current pruritus treatment; other skin disorders; malignancy; malabsorption; kidney transplantation; INR >1.1; PTH >300 pg/mL or phosphorus levels >7 mg/dL; hemoglobin <10 g/dL
Shayanpour et al. [34]	Iran	Age >18 years old with HD duration >3 months	Liver diseases; skin diseases; malignancy; allergy to omega-3 supplements; kidney transplantation
Heydarbaki et al. [35]	Iran	>18 years; no drinking alcohol; conscious; diagnosis of CRF ≥4 weeks; HD for ≥2 sessions per week; chronic itching ≥4 weeks	Taking other antipruritics; death during study, other skin diseases causing itching; contraindication of antihistamines; taking any anti-itching drug except cetirizine; topical treatments which can induce itching; sensitivity to omega-3; digestive problems
Forouhari et al. [36]	Iran	ESRD patients; age >18 years; intermittent HD >3 months; pruritus >8 weeks; no history of pruritic skin disease before renal failure	Other skin diseases; hepatic cholestasis; hepatitis B/C; steroid treatment; current treatment for uremic pruritus; malabsorption; malignancy; kidney transplantation; anemia; phosphorus level >7 mg/dL; PTH >300 μg/L); anticoagulants; allergic reaction; GIT problems
Rafieipoor et al. [37]	Iran	Consent; age >20 years; KT/V higher than the standard range; no omega-3 fatty acid supplement during the last three months; no history of PD; no surgery in the previous six months; no allergy to omega-3, medium chain triglycerides oil, or fish and fish products; not pregnant	Psychiatric conditions; intellectual disability; active inflammatory, infection, pulmonary, cardiac, hemoglobinopathies and coagulopathy conditions; malignancy; recent use of immunosuppressant, chemotherapeutics or anticoagulants; NSAIDs; corticosteroids; non-compliance with omega-3 supplementation or HD; disease aggravation; hospitalization/surgery

The sample size showed some variations in the studies, ranging from eight patients per group and up to 58 per group. The control was safflower oil in two studies [12,13,31] and placebo in five studies [32-34,36,37]. One study used cetirizine in the intervention and control groups as it was the standard treatment for uremic pruritus in their institution, with omega-3 administered as an adjuvant [35]. The studies were comparable in the mean age of included patients, while the sex distribution varied slightly (Table 2).

**Table 2:** Summary of baseline criteria in the included studies (n = 8) F: female; M: male; FO: fish oil; OO: olive oil; SD: standard deviation; SO: safflower oil

Study	Sample size		Age (years), mean ± SD		Gender (M:F)	
	Omega-3	Control	Omega-3	Control	Omega-3	Control
Peck et al. [12,13]	FO: 8	OO: 9; SO: 8	54.8 ± 16.2	OO: 45.6 ± 17.4; SO: 49.5 ± 17.2	5:3	OO: 4:5; SO: 4:4
Begum et al. [31]	FO: 12	SO: 10	61.2 ± 19.42	49.25 ± 18.12	7:3	6:6
Ghanei et al. [32]	11	Placebo: 11	59.90 ± 14.82	53.09 ± 13.08	72%:28%	54%:46%
Lahiji et al. [33]	20	Placebo: 20	62.1 ± 11.6	61.9 ± 10.8	9:11	10:10
Shayanpour et al. [34]	32	Placebo: 32	51.91 ± 6.586	56.25 ± 8.865	27:5	23:9
Heydarbaki et al. [35]	26	Cetirizine: 26	61.34 ± 15.7		57.7%:42.3%	
Forouhari et al. [36]	17	Placebo: 16	59.00 ± 13.56	51.25 ± 15.85	70.6:29.4	68.8:31.2
Rafieipoor et al. [37]	58	Placebo: 54	61.17 ± 12.35	55.33 ± 12.6	30:28	37:17

Four studies had a cross-over design [32,33,35,36]. Omega-3 oil was administered as oral capsules in all studies, with varying dosages and durations. Assessment of pruritus was done using the Duo questionnaire in three studies [12,13,31,32] and the VAS in three studies [33,35,36]. One study used the 5-D itch scale [34], while another study used the Worst Itching Intensity Numerical Rating Scale (Table 3) [37].

**Table 3:** Arms and method of assessing pruritus in the included studies (n = 8). VAS: visual analog scale; WI-NRS: Worst Itching Intensity Numerical Rating Scale

Study	Omega-3 form/route/dose	Control	Pruritus assessment tool
Peck et al. [12,13]	Soft-gel capsules containing 1 g fatty acid ethyl ester (National Oceanic and Atmospheric Administration, Charleston Laboratory). Dose: six capsules/day for eight weeks	SA: soft-gel capsules containing 1 g fatty acid ethyl ester (National Oceanic and Atmospheric Administration, Charleston Laboratory). Dose: six capsules/day for eight weeks	Duo questionnaire
Begum et al. [31]	Soft-gel capsules containing 1 g fish oil fatty acid ethyl ester (National Oceanic and Atmospheric Administration, Charleston Laboratory). Dose: six capsules/day for 16 weeks	Soft-gel capsules containing 1 g fatty acid ethyl ester (National Oceanic and Atmospheric Administration, Charleston Laboratory). Dose: six capsules/day for 16 weeks	Duo questionnaire
Ghanei et al. [32]	Fish oil 1 g capsules (Zahravi, Tabriz, Iran) three capsules/day for 20 days. After a 14-day washout period, they were treated with a placebo for 20 days	Identical placebo capsules for 20 days. After a 14-day washout period, they were treated with omega-3 for 20 days	Duo questionnaire
Lahiji et al. [33]	Three 1 g omega-3 capsules (Zahravi, Tabriz, Iran) per day for one month. After a washout period of six weeks, patients were crossed over to the alternate treatment	Three 1 g placebo capsules (Zahravi, Tabriz, Iran) per day for 1 month. After a washout period of six weeks, patients were crossed over to the alternate treatment	VAS
Shayanpour et al. [34]	A single dose of 2 g omega-3 capsules daily before lunch for three weeks	A single dose of placebo capsules three daily before lunch for three weeks	5-D itch scale
Heydarbaki et al. [35]	1 g of omega-3 three times a day + 5 mg cetirizine three times a week for six weeks. After a washout period of one week, groups changed the treatments	5 mg of cetirizine three times a week for six weeks. After a washout period of one week, groups changed the treatments	VAS
Forouhari et al. [36]	Omega-3 1 g capsules (Zahravi, Tabriz, Iran) three times a day for four weeks. After a six-week washout period, a placebo is received for four weeks	Placebo capsules (Zahravi, Tabriz, Iran) three times a day for four weeks. After a six-week washout period, omega-3 is received for four weeks	VAS
Rafieipoor et al. [37]	Three 1 g capsules of omega-3 fatty acids/day (Zahravi Pharmaceutical Co, Tabriz, Iran) for two months	Three placebo capsules containing medium-chain triglyceride (Zahravi Pharmaceutical Co, Tabriz, Iran) for two months	WI-NRS

Assessment of the Risk of Bias in the Included Studies

The ROB was assessed using the ROB2 tool which includes five domains besides an assessment of the overall risk (Table 4). The ROB regarding the process of randomization was low in three studies [34,35,37]. Four studies showed some concerns due to a lack of details about the generation of the random sequence [31,32,36] and allocation concealment [12,13,31,32,36]. Moreover, one study had a high ROB due to a lack of details about randomization and concealment, as well as a significantly higher mean VAS score in the omega-3 group [33]. The ROB arising from deviations from intended interventions was low in all studies except for one study due to lack of clarity about 16 patients who were randomized but did not complete the intervention (Table 4) [12,13]. The risk of missed outcome data was low in all studies, except in two where the risk was high due to the high number of missed data and no information about the distribution of lost to follow-up patients in both groups [12,13,36]. Measurement of the outcome showed low ROB in all studies. There was some risk of selective reporting of outcomes in five studies [12,13,31-33,36], as no protocol was available to compare the methodology reported in the protocol and published article (Table 4).

**Table 4:** The risk of bias assessment for the included trials based on the ROB2 tool (n = 8). D1: randomization process; D2: deviations from intended interventions; D3: missing outcome data; D4: measurement of the outcome; D5: selection of the reported result

Study	D1	D2	D3	D4	D5	Overall
Peck et al. [12,13]	Some concerns	High	High	Low	Some concerns	High
Begum et al. [31]	Some concerns	Low	Low	Low	Some concerns	Some concerns
Ghanei et al. [32]	Some concerns	Low	Low	Low	Some concerns	Some concerns
Lahiji et al. [33]	High	Low	Low	Low	Some concerns	High
Shayanpour et al. [34]	Low	Low	Low	Low	Low	Low
Heydarbaki et al. [35]	Low	Low	Low	Low	Low	Low
Forouhari et al. [36]	Some concerns	Low	High	Low	Some concerns	High
Rafieipoor et al. [37]	Low	Low	Low	Low	Low	Low

Results of the Meta-Analysis

Post-intervention pruritus score: Six studies reported the score of pruritus severity after intervention in both groups [13,31,32,34-36]. Four studies reported a significantly lower mean score of pruritus severity in the omega-3 group compared to the control group [32,34-36]. Meanwhile, the other two studies found a lack of significant difference between the two groups (Table 5) [13,31].

**Table 5:** Summary of the scores for assessing pruritus. The table presents the summary descriptive statistics for the scores used to assess pruritus. The scores varied across the studies as described in the Results section. Some studies reported the scores for assessing the severity of the pruritus, while others reported the results of subscales of the scores assessing the distribution and frequency of pruritus as well as awakening from sleep because of itching. The table provides the results for the scales of severity and the other subscales as reported by the studies. The numbers represent the mean and standard deviation of the score (one study reported the 95% confidence interval instead of the standard deviation). The scores do not provide a unit of measurement (these are the mean and standard deviation for the sum of points from the points assigned to each item in the scale). values are presented as mean ± standard deviation unless reported otherwise. CI: confidence interval; NR: not recorded; OO: olive oil; SO: safflower oil

Score items and studies	Baseline		Post-intervention		Change	
	Omega-3	Control	Omega-3	Control	Omega-3	Control
Severity of pruritus						
Peck et al. [12,13]	3.25 ± 1.58	OO: 2.33 ± 1.32; SO: 2.87 ± 1.25	1.63 ± 1.41	OO: 1.56 ± l.33; SO: 3.0 ± 1.77	-1.63 ± 1.77	-0.78 ± 0.97; 0.13 ± 1.64
Begum et al. [31]	2.8 ± 1.2	2.3 ± 1.2	1.7 ± 1.1	1.7 ± 0.4	-1.1 ± 1.5	-0.5 ± 1.1
Ghanei et al. [32]	20.3 (95% CI = 16.7–23.8)	17 (12.4–21.6)	6.4 (2.9–9.8)	14.4 (10.5–18.2)	NR	NR
Lahiji et al. [33]	6.4 ± 1.5	4.2 ± 2.4			-3.02 ± 1.8	-0.48 ± 1.4
Shayanpour et al. [34]	3.56 ± 0.669	3.63 ± 0.609	1.72 ± 0.634	3.09 ± 0.963	NR	NR
Heydarbaki et al. [35]	6.40	6.33	2.90	4.61	NR	NR
Forouhari et al. [36]	6.59 ± 2.45	5.81 ± 3.17	3.18 ± 2.51	5.87 ± 2.25	-3.41 ± 2.62	0.06 ± 2.54
Distribution of pruritus						
Peck et al. [13]	1.88 ± 0.99	OO: 2.33 ± 0.71; SO: 2.13 ± 0.64	1.25 ± 1.04	OO: 1.22 ± 097; SO: 1.88 ± 0.99	-0.63 ± 0.52	-1.11 ± 1.05; -0.25 ± 0.46
Begum et al. [31]	2.3 ± 0.6	2.6 ± 0.7	1.5 ± 1.1	2.2 ± 0.8	-0.8 ± 1.1	-0.4 ± 0.8
Frequency of pruritus						
Peck et al. [13]	3.66 ± 4.27	OO: 1.97 ± 2.40; SO: 3.18 ± 4.27	2.07 ± 3.62	OO: 0.84 ± 1.36; SO: 3.18 ± 4.41	-1.59 ± 3.51	OO: -1.13 ± 0.57; SO: 0.01 ± 1.01
Begum et al. [31]	3.7 ± 2.2	5.5 ± 2.4	2.2 ± 2.0	4.6 ± 2.5	-1.5 ± 2.3	-0.9 ± 2.8
Awakening to itching						
Begum et al. [31]	4.1 ± 5.1	3.3 ± 3.9	1.4 ± 3.1	1.2 ± 0.9	-2.7 ± 4.8	-2.1 ± 3.8

**Figure 2:**
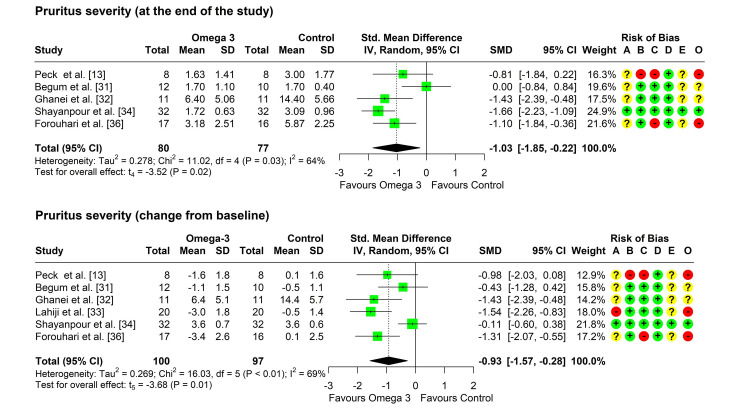
Forest plot showing pooling of the studies’ findings regarding the pruritus severity at the end of treatment and change from baseline. CI: confidence interval; SD: standard deviation; SMD: standardized mean difference; A: randomization process; B: deviations from intended interventions; C: missing outcome data; D: measurement of the outcome; E: selection of the reported result; O: overall risk of bias

As one study did not report the standard deviations of the severity score before or after treatment, this study was not included in the meta-analysis [35]. Heterogeneity testing was significant (chi-square = 11.02, p = 0.026, I^2^ = 64%), so the random-effects model was used to pool the results. The pooled SMD (95% CI) was -1.03 (-1.85, -0.22), with a p-value of 0.024 (Figure 2). No outliers were detected but leave-one-out analysis suggested that the study by Begum et al. may be influential as its omission increased the pooled SMD to -1.35 (-1.94, -0.77) and reduced the I^2^ index to 0% [31].

Change in severity of pruritus from baseline: Six studies reported a change in the severity of pruritus from baseline in both groups [13,31-34,36]. Heterogeneity testing was significant (chi-square = 16.03, p = 0.007, I^2^ = 69%), so the random-effects model was used to pool the results. The pooled SMD (95% CI) was -0.93 (-1.57, -0.28), with a p-value of 0.014 (Figure 2). No outliers were detected but leave-one-out analysis suggested that the study by Shayanpour et al. may be influential as its omission increased the pooled SMD to -1.17 (-1.74, -0.61) and the I^2^ index to 11% [34].

**Figure 3:**
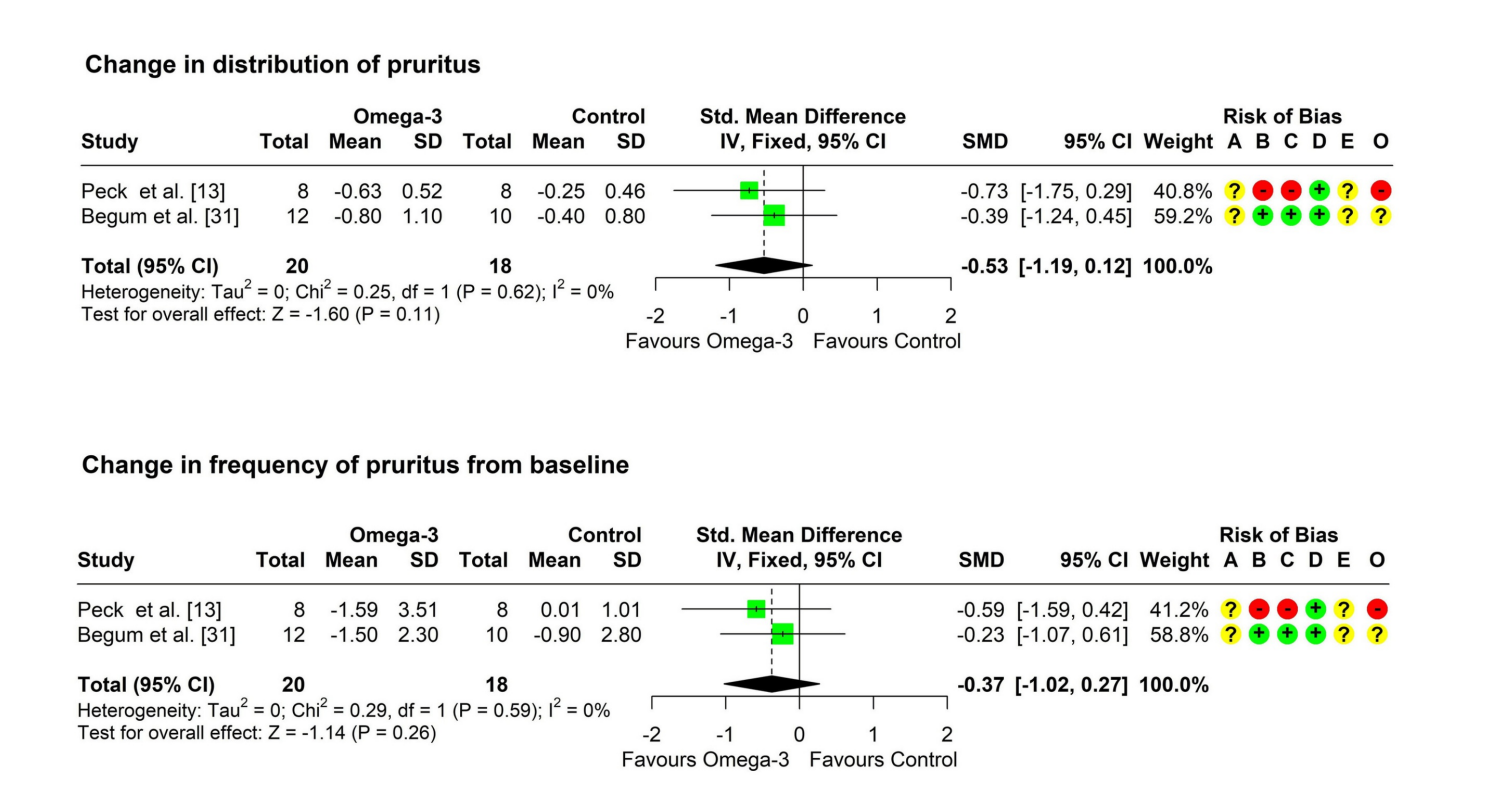
Forest plot showing pooling of the studies’ findings regarding the change from baseline in the distribution and frequency of pruritus. CI: confidence interval; SD: standard deviation; SMD: standardized mean difference; A: randomization process; B: deviations from intended interventions; C: missing outcome data; D: measurement of the outcome; E: selection of the reported result; O: overall risk of bias

Change in distribution of pruritus from baseline: Two studies reported a change in the distribution of pruritus from baseline in both groups [13,31]. Heterogeneity testing was significant (chi-square = 0.25, p = 0.618, I^2^ = 0%), so the fixed-effect model was used to pool the results. The pooled SMD (95% CI) was -0.53 (-1.19, 0.12), with a p-value of 0.111 (Figure 3).

Change in frequency of pruritus from baseline: Two studies reported a change in the frequency of pruritus from baseline in both groups [13,31]. Heterogeneity testing was significant (chi-square = 0.29, p = 0.593, I^2^ = 0%), so the fixed-effect model was used to pool the results. The pooled SMD (95% CI) was -0.37 (-1.02, 0.27), with a p-value of 0.256 (Figure 3).

**Figure 4:**
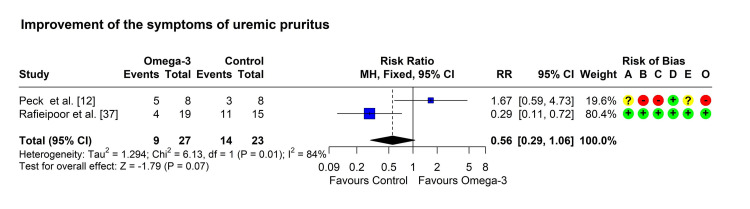
Forest plot showing pooling of the studies’ findings regarding the improvement of pruritic symptoms. CI: confidence interval; MH: Mantel-Haenszel method; RR: risk ratio; A: randomization process; B: deviations from intended interventions; C: missing outcome data; D: measurement of the outcome; E: selection of the reported result; O: overall risk of bias

Improvement of the symptoms of pruritus: Two studies reported an improvement in the symptoms of pruritus in both groups [12,37]. One study reported a significantly higher percentage of improvement in the omega-3 group [12], while the other study found a non-significantly higher percentage of improved patients in the control group [37]. Heterogeneity testing was significant (chi-square = 6.13, p = 0.013, I^2^ = 84%), so the random-effects model was used to pool the results. The pooled RR (95% CI) was 0.68 (0.12, 3.81), with a p-value of 0.661 (Figure 4).

Adverse effects: Only two studies mentioned the results of recording intervention-related side effects. One study reported that all patients tolerated omega-3 and no side effects were detected after treatment with 2 g of omega-3 for three weeks [34]. Meanwhile, the second study found that 9% of patients using omega-3 complained of mild gastrointestinal symptoms (e.g., nausea and diarrhea) [36].

Discussion

Summary of the Main Findings

A considerable proportion of ESRD patients suffer from CKD-aP, with severe and negative impacts on their quality of life. Previous studies reported that ESRD patients exhibited abnormal fatty acid profiles. In addition, ESRD patients suffer symptoms that may be attributed to the deficiency of essential fatty acids, including pruritus, abnormal sweating, delayed wound healing, anemia, and liability to hemolysis. This deficiency of omega-3 fatty acids in CKD patients could be explained by the dietary restrictions in renal patients, the lack of issued recommendations on enhancing fish ingestion, and socioeconomic factors that may limit the purchase and consumption of dietary sources such as fish or supplements [10].

Supplementation with essential fatty acids and their derivatives may theoretically provide several health benefits to those patients by correcting the deficiency of essential fatty acids. The present systematic review and meta-analysis aimed to summarize the evidence regarding the efficacy and safety profile of omega-3 supplementation in the treatment of CKD-aP. The search and selection strategies yielded eight eligible studies which were included in this review.

We found that treatment with omega-3 fatty acids showed a significantly lower severity of CKD-aP at the end of treatment (pooled SMD (95% CI) = -1.03 (-1.85, -0.22), p = 0.024 and change from baseline (pooled SMD (95% CI) = -0.93 (-1.57, -0.28), p = 0.014). The efficacy of omega-3 fatty acid supplements could be explained by their anti-inflammatory effect [38].

Peck et al. proposed that omega-3 fatty acids block the lipoxygenase pathway [12,13], resulting in the lowering of arachidonic acid concentration, increasing prostaglandin E2 (PGE2) concentration, and shifting the metabolism of arachidonic acid to the cyclooxygenase pathway. The net result of these effects is reduced release of inflammatory products (e.g., PGE2) and decreased severity of pruritus. The authors also hypothesized that omega-3 fatty acids enhance the synthesis of the series-3 eicosanoids, which exert less inflammatory activity. Increased series-3 eicosanoids concentrations may prevent the synthesis of the series-2 eicosanoids [38].

Moreover, Begum et al. found that fish oil and safflower oil decreased the synthesis of leukotriene B4 by polymorphonuclear leukocytes [31]. However, this reduction was significantly lower with fish oil administration compared to safflower oil.

Three studies out of the included eight showed a lack of significant difference in improving CKD-aP between the omega-3 and control groups. The studies showed variations in several aspects which presumably contributed to the observed significant heterogeneity. The regimen of omega-3 supplements and the assessment tool for the severity of pruritus varied across the studies.

In addition, there were differences in the control group as those three studies used another type of fatty acid as a control. Peck et al. [12,13], and Begum et al. used safflower oil [31], which showed improvement in pruritus compared to baseline, though lower than that of the omega-3 fatty acids. Rafieipoor et al. used medium-chain triglycerides (MCTs) as a placebo for the control group [37]. The rate of CKD-aP was comparably decreased in both groups, suggesting that MCTs may similarly exert a beneficial effect in treating CKD-aP. The antipruritic effect of omega-3 fatty acids may differ based on the source of fatty acids (animal or vegetable) [39,40], or their composition [41].

We found that only two studies recorded the occurrence of intervention-related adverse effects. The effects were minimal and presented as mild gastrointestinal disturbances. This accords with the excellent safety profile of omega-3, as reported by the American Heart Association [42]. Omega-3 fatty acid administration at the recommended dose (1 g EPA and DHA) per day has not been associated with serious adverse effects; therefore, it can be safely prescribed in advanced CKD patients [10]. Nevertheless, longitudinal studies are required to assess the safety profile of omega-3 supplementation in ESRD patients after a long duration of consumption.

Previous meta-analyses reported nearly similar findings, though they included fewer studies than this meta-analysis. Yeam et al. [17], and Lu et al. assessed five studies [43]. The former systematic review summarized the controversial results of the studies while the latter pooled the results and found that the pruritus score did not significantly decrease after supplementation with omega-3 fatty acids compared to the control group (SMD = 1.34, 95% CI = -2.70 to 0.01, p = 0.05) [17,43]. A more recent meta-analysis by Boehlke et al. included only four studies and reported that fish oil/omega-3 fatty acids supplementation may lead to a large decrease in the severity of pruritus (SMD = -1.60, 95% CI = -1.97 to -1.22) compared to the placebo [44]. The differences in the results of the conducted meta-analyses can be explained by the differences in the included studies.

Overall Completeness, Applicability, and Quality of the Evidence

The evidence summarized in this systematic review and meta-analysis suggests that omega-3 fatty acid supplementation can improve CKD-aP. However, the included studies showed several limitations which affect the quality of evidence. The duration of omega-3 intake was short in all studies and was two months or less, except in the study by Begum et al. [31]. In addition, five of the included studies raised concerns regarding the potential for some or high ROB in different domains.

Another potential source of concern is the safety of long-term administration of omega-3 fatty acids in ESRD patients. The currently available research did not provide evidence for this due to the short duration of studies and the non-reporting of adverse effects, except for two studies. Meanwhile, as research reported several potential health benefits of omega-3 in patients with advanced CKD, including the improvement of dyslipidemia, hypertension, cardiovascular diseases, immune response, inflammation, and reduced risk of all-cause mortality [45-47], future guidelines should consider recommending the use of omega-3 in CKD patients using the doses recommended by the American Heart Association [42].

## Conclusions

Omega-3 fatty acid supplementation appears to be a promising, effective, and safe line of treatment for CKD-aP. However, the included studies exhibited several limitations, which necessitates further high-quality studies to elucidate its effect and investigate the causes for non-response in patients who did not improve.
